# Inter-hemispheric integration of tactile-motor responses across body parts

**DOI:** 10.3389/fnhum.2015.00345

**Published:** 2015-06-15

**Authors:** Luigi Tamè, Matthew R. Longo

**Affiliations:** Department of Psychological Sciences, Birkbeck, University of LondonLondon, UK

**Keywords:** tactile, body parts, interhemispheric transfer, CUD, sensory-motor integration

## Abstract

In simple detection tasks, reaction times (RTs) are faster when stimuli are presented to the visual field or side of the body ipsilateral to the body part used to respond. This advantage, the *crossed-uncrossed difference* (CUD), is thought to reflect inter-hemispheric interactions needed for sensorimotor information to be integrated between the two cerebral hemispheres. However, it is unknown whether the tactile CUD is invariant when different body parts are stimulated. The most likely structure mediating such processing is thought to be the corpus callosum (CC). Neurophysiological studies have shown that there are denser callosal connections between regions that represent proximal parts of the body near the body midline and more sparse connections for regions representing distal extremities. Therefore, if the information transfer between the two hemispheres is affected by the density of callosal connections, stimuli presented on more distal regions of the body should produce a greater CUD compared to stimuli presented on more proximal regions. This is because interhemispheric transfer of information from regions with sparse callosal connections will be less efficient, and hence slower. Here, we investigated whether the CUD is modulated as a function of the different body parts stimulated by presenting tactile stimuli unpredictably on body parts at different distances from the body midline (i.e., Middle Finger, Forearm, or Forehead of each side of the body). Participants detected the stimulus and responded as fast as possible using either their left or right foot. Results showed that the magnitude of the CUD was larger on the finger (~2.6 ms) and forearm (~1.8 ms) than on the forehead (≃0.9 ms). This result suggests that the interhemispheric transfer of tactile stimuli varies as a function of the strength of callosal connections of the body parts.

## Introduction

Exchange of information between the two hemispheres is a fundamental function by which signals from the two sides of the body are integrated, allowing coherent perception and coordinated action. At the beginning of last century, Poffenberger was the first to behaviorally quantify this sensorimotor transfer in a series of seminal experiments in the visual domain (Poffenberger, 1912; Marzi, 1999). He showed that people have faster reaction times (RTs) when visual stimuli are presented in the visual field ipsilateral to the hand used to respond. He proposed that this *crossed-uncrossed difference* (CUD) reflects the time required for signals to transfer between the two cerebral hemispheres. This *inter-hemispheric transfer time* (IHTT) of sensorimotor information has been estimated to be between about 2–6 ms (Poffenberger, 1912; Marzi et al., 1991; Aboitiz et al., 1992; Berlucchi et al., 1995; Pellicano et al., 2013).

The logic of the Poffenberger paradigm is that when the sensory stimulus and motor effector are on the same side of the body, sensorimotor information can be integrated and processed within the same hemisphere (uncrossed time). By contrast, if sensory input is presented contralateral to the effector used to respond, the information has to be integrated across hemispheres (crossed time). Poffenberger and many subsequent researchers argued that the most likely anatomical pathway to mediate the effect is the corpus callosum (CC; Poffenberger, 1912; Marzi et al., 1991; Berlucchi et al., 1995). This interpretation is strongly supported by studies on acallosal patients, who show significantly slower RTs in the crossed compared to the uncrossed condition (between 12–27 ms; Milner et al., 1985; Aglioti et al., 1993).

Other evidence, however, has called this model into question. For example, estimation of the effect from electrophysiological data has indicated a longer estimation of the IHTT compared to simple RTs (Brown et al., 1994; Meyer et al., 1995). Moreover, neural signals and RTs appear to be uncorrelated (Saron and Davidson, 1989). For these reasons, some authors have suggested that the CUD cannot be considered a pure measure of IHTT (Braun et al., 2003) if other sources of variation (e.g., uncertainty of location, S-R compatibility, specialized cognitive processing) are not held constant (Kinsbourne, 2003). However, there is a general agreement that simple RT tasks activate multiple parallel callosal and subcallosal channels that mediate the transferring of sensory, motor and/or cognitive information (Zaidel and Iacoboni, 2003). Furthermore, it has been proposed that the dominant channel vary or switch as a function of the task demand.

Most studies investigating the CUD effect have been in vision (Jeeves, 1969; Bashore, 1981; Marzi et al., 1991; Pellicano et al., 2013; Chaumillon et al., 2014), with only a few investigating other sensory modalities such as touch (Muram and Carmon, 1972; Moscovitch and Smith, 1979; Schieppati et al., 1984; Kaluzny et al., 1994), audition (Elias et al., 2000; Böhr et al., 2007), and cross-modally (Tassinari and Campara, 1996). Fendrich et al. (2004) directly investigated the CUD in vision and touch, showing that its magnitude is comparable in the two sensory modalities.

However, despite numerous studies on healthy subjects (Jeeves, 1969; Berlucchi et al., 1971; Tettamanti et al., 2002; Fendrich et al., 2004; Pellicano et al., 2013; Chaumillon et al., 2014) and patients (Volpe et al., 1982; Savazzi et al., 2008), it is unknown whether in the tactile modality the CUD is modulated as a function of the body part stimulated. In vision it has been shown that the CUD does not vary when either luminance (Forster and Corballis, 1998) or eccentricity (Berlucchi et al., 1971, 1995; Aglioti et al., 1991) is modulated. The logic of this approach is that if the CUD reflects, at least in part, transferring of the sensory information, stimuli presented on regions of the visual field with few or no callosal connections should produce a greater CUD compared to stimuli presented on other portions of the visual field with denser callosal connections. This is because the sensorimotor interhemispheric transfer from regions with sparse callosal connections will be less efficient, and hence slower. Studies that manipulated the eccentricity of the visual stimuli found no modulatory effect on the CUD.

Other evidence, however, has suggested that the CUD might be affected by the callosal connections of the sensory regions. For example, in a study of a patient with a lesion of the CC, but with an intact splenium (i.e., posterior part of the CC), Tassinari et al. (1994) found no increase in the CUD. As the splenium is thought to mediate the transferring of visual rather than motoric information, the authors suggested that the CUD could reflect the interhemispheric transfer of both sensory and motor signals (Bisiacchi et al., 1994; Tassinari et al., 1994).

Here, we investigated whether tactile stimuli delivered on different parts (i.e., Finger, Arm, Forehead) of the two sides of the body, produce comparable CUDs, or whether instead they produce a different CUD as a function of the body part stimulated. Neurophysiological studies have shown that callosal connections are denser between regions of the two hemispheres that represent proximal parts of the body near the body midline than more distal extremities (Manzoni et al., 1989). If the callosal connections between the different parts of the two sides of the body affect the sensorimotor interactions determining the CUD, the magnitude of the CUD should be reduced or absent on proximal, compared to distal, parts of the body. Instead, if the callosal connections do not affect sensorimotor interactions, the CUD should be similar, whichever body part is stimulated.

Neurophysiological studies in monkeys have found that callosal connections between the different parts of the two side of the body are more dense for the most proximal regions (e.g., trunk, face) and sparser, although clearly present, for the most distal regions such as hands and fingers (Killackey et al., 1983; Iwamura, 2000; Lipton et al., 2006). Similarly, neuroimaging studies in humans have shown that unilateral tactile stimulation can elicit activity in the ipsilateral primary sensory cortex (Polonara et al., 1999; Malinen et al., 2014) and in multiple regions of the CC (Fabri et al., 2011). The logic of our experiment is based on the relative difference in the density of the callosal connections between the different body regions. Specifically, on the relative reduction of such connections from the medial to the more distal regions of the body (Killackey et al., 1983; Iwamura, 2000).

## Material and Methods

### Participants

Twenty-eight participants (mean ± SD = 28.9 ± 8.0 years; 22 females) took part in the study. Participants gave their informed consent prior to participation and reported normal or corrected to normal vision and normal touch. The study was approved by the local ethics panel. All participants but two were right-hand, as assessed by the Edinburgh Handedness Inventory (Oldfield, 1971; M = 71, range: −100 to 100).

### Apparatus and Stimuli

Tactile stimuli were delivered on the middle fingers of both hands, on the arms and on two locations on the forehead using six stimulators (Solenoid Tactile Tapper, M&E Solve, UK). The solenoid tappers (8 mm in diameter) producing the suprathreshold tactile stimuli were driven by a 9 V square wave. The apparatus was controlled by means of a National Instrument Card (NI USB-6341) connected to a PC through a USB port. Tactile stimulation was delivered for 5 ms. All participants clearly perceived this stimulation when delivered in isolation to each body part before the experiment. To ensure that when in operation the stimulators produced an equal force to the fingers, a piezoelectric pressure sensor (MLT1010, AD Instruments, Dunedin, New Zealand) was used to measure the intensity of each tapper. Furthermore, tappers assigned to each body part (left or right middle finger, arm and forehead) were randomly changed for every participant, to control for undetectable intensity differences between the stimulator devices.

**Figure 1:**
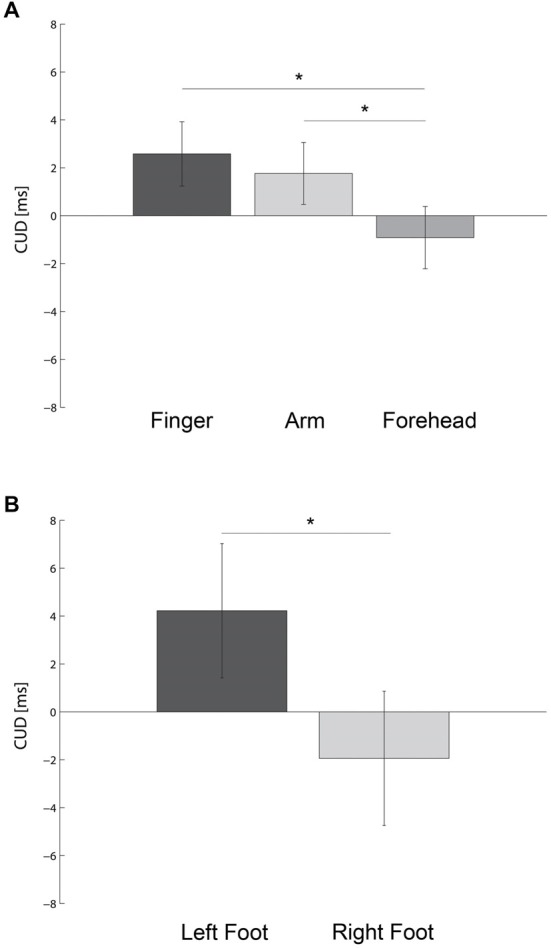
**Bar plots show the crossed-uncrossed difference (CUD) as a function of the body part stimulated (A) and foot used to respond (B)**. Error bars indicate the 95% Confidence Interval of the within participants variability (±CI). * denotes *P* < 0.05.

Tactile stimulators were attached on the body parts using double-sided adhesive collars (ADD204 19 mm OD, 4 mm ID). The hands rested on the table 20 cm apart from one another (see Figure 1). In this way, the stimulators exerted a similar pressure on all body parts. Two tactile stimulators were positioned on the center of the most distal phalanx of the middle fingers. Two other stimulators were positioned 2 cm towards the wrist from the antecubital fossa (i.e., the inside of the arm at the elbow) and centered with respect to the arms’ width (~30 cm far apart). Finally, the last two stimulators were positioned centrally on the vertical plane between the nasion and the hairline and on the horizontal plane 1 cm apart from the body midline of the forehead.

Vision of the limbs was prevented throughout by means of a sheet of black cardboard, placed horizontally on a structure fixed to the table, on top of the hands. One foot-response pedal was positioned under the participant’s feet aligned with his body midline. In order to prevent potential confounds of a compatibility effect due to the sensorimotor interactions we have chosen distant stimulation (i.e., upper body parts) and response (i.e., feet) locations (Broadbent and Gregory, 1965; Fendrich et al., 2004). In this respect, it has been recently shown that stimulus response compatibility effects under bimanual reaction times to lateralized visual stimuli cannot account for CUD (Pellicano et al., 2013). Stimulus presentation and response collection were controlled by a custom program written using MATLAB R2013b (Mathworks, Natick, MA) and Psychtoolbox libraries (Brainard, 1997). Throughout the experiment, white noise was presented over closed-ear headphones (Sennheiser HD 439 Audio Headphones) to mask any sounds made by the tactile stimulators.

### Design

The experiment followed a repeated-measures design with three factors. These were BODY PART, which includes the stimulation of the fingers, arms or forehead, response FOOT that was the left or the right and SIDE that was crossed or uncrossed representing the compatibility between side of stimulation and response. Participants underwent a series of 10 blocks in which they were asked randomly in half of them to respond with the left foot and the other with the right foot. Blocks were randomized with respect to the sides and body parts, whereas the response foot was blocked. Each block comprised 120 trials, resulting in a total of 1200 trials for each participant.

### Procedure

Before the main experiment, each participant performed about 40 practice trials to familiarize them with the task, assure they could clearly perceive the stimuli equally on all the body parts and that tactile stimuli were not audible. Participants were informed that they had to respond as quickly as possible as soon as they felt a tactile stimulus on one of the body parts with their foot. On each trial only one body part was tactile stimulated. Participants were instructed to keep the foot-pedal pressed, unless indicating the occurrence of the stimulus by raising it. Participants were instructed to keep their gaze centered in front of them to a black sticker attached to the wall aligned with their body midline to control for head (Ho and Spence, 2007) and gaze (Harrar and Harris, 2009; Gherri and Forster, 2014) positions. At the beginning of each trial after a variable interval, ranging from 1000 to 2000 ms a tactile stimulus was presented. Participants were allowed short breaks between blocks. The experimenter remained in the room throughout the session to ensure that participants complied with the instructions.

### Data Analysis

Responses shorter than 100 ms were considered anticipations and responses over 500 ms were considered attentional errors (Iacoboni and Zaidel, 2000). Trials excluded were rerun at the end of each block to assure the same number of trials for each condition (Fendrich et al., 2004), except for the first five participants due to a technical fault (average number of trials lost M ± SE = 10% ± 2.6). The overall number of rerun trials was M ± SE = 5.7% ± 0.9. For each participant, we computed the CUD by subtracting the RTs in the uncrossed from the RTs in the crossed conditions for the different body parts. A negative CUD indicates that participants were faster in responding when stimulation and response side were the same, whereas a positive CUD indicates that participants were faster in responding when stimulation and response side were different. The CUDs values were entered into a two-way Analysis of Variance (ANOVA) with BODY PART (Finger, Arm, and Forehead) and FOOT (Left, Right) as within-participant factors. Two-tailed paired *t*-tests were used for all planned comparisons.

## Results

An ANOVA on CUD values revealed a significant main effect of BODY PART, *F*_(2,54)_ = 4.97, *p* < 0.0001, MSE = 37.7, η_*p*_^2^ = 0.16. As shown in Figure 1A, when stimuli were presented on the forehead (M ± SE = −0.92 ± 0.8 ms) there was a significantly lower CUD compared to the forearms (*M* = 1.76 ± 1.2 ms, *t*_(27)_ = 2.36, *p* < 0.03, *d*_z_ = 0.45) and fingers (*M* = 2.58 ± 1.1 ms, *t*_(27)_ = 2.97, *p* < 0.006, *d*_z_ = 0.56). However, the CUD value did not differ between forearms and fingers (*t*_(27)_ = 0.70, *p* = 0.49, *d*_z_ = 0.13). On the fingers the CUD was significantly different from zero (*t*_(27)_ = 2.29, *p* < 0.03, *d* = *0*.43). The CUD did not differ significantly from 0 on either the forearm (*t*_(27)_ = 1.53, *p* = 0.14, *d* = *0*.29) or forehead (*t*_(27)_ = 1.20, *p* = 0.24, *d* = *0*.23).

In addition, a significant main effect of FOOT was also present, *F*_(1,27)_ = 4.64, *p* < 0.04, MSE = 344.1, η_*p*_^2^ = 0.15. As shown in Figure 1B, when participants responded with the left foot they had a significant positive CUD effect (CUD = 4.2 ± 1.8 ms). In contrast, when participants responded with their right foot, there was a tendency towards a negative CUD (CUD = −1.9 ± 1.5 ms). This marked asymmetry in the CUD replicates results from previous reports when hands were used as effectors (Marzi et al., 1991; Kaluzny et al., 1994; Fendrich et al., 2004).

## Discussion

This study investigated whether the interhemispheric transfer of tactile stimuli is modulated as a function of the body part stimulated. To this end we presented tactile stimuli unpredictably on different parts of the two sides of the body, namely the middle fingers, arms and forehead. We found that the CUD was significantly greater when participants were stimulated on the fingers or forearm, compared to when they were stimulated on a more proximal body part, such as the forehead. The differences in CUD magnitude between distal (fingers, forearm) and proximal (forehead) regions is compatible with the distribution of the callosal connections and the density of bilateral receptive fields (RFs) between the regions that represent the body from the periphery to the center (Pandya and Vignolo, 1969; Caminiti and Sbriccoli, 1985; Iwamura et al., 2001).

The CUD was strong when participants responded with the left foot, but absent when they responded with the right foot. This is in agreement with a meta-analysis of 16 studies in which Marzi et al. (1991) showed a marked asymmetry of the CUD towards the left hand (i.e., the effector used to respond). More recently, Fendrich et al. (2004) found that the same asymmetry was present in both vision and touch.

### Tactile Interhemispheric Transfer Varies Across the Body

The presence of a CUD when we delivered stimuli on the fingers is consistent with previous reports investigating inter-hemispheric transfer in touch (Muram and Carmon, 1972; Moscovitch and Smith, 1979; Fendrich et al., 2004) as well as in vision (Bashore, 1981; Marzi et al., 1991). Differently from vision, we found that the CUD was modulated as a function of the spatial position of the stimuli (i.e., the body part stimulated). In particular, we found a CUD of 2.6 ms when the fingers, but not other body parts (i.e., arm and forehead) were stimulated. This pattern of results is compatible with the conduction capability of the CC (Aboitiz et al., 1992; Caminiti et al., 2013). Although there are relatively few reports in the tactile domain, previous studies have estimated a tactile CUD between 2–17 ms (Muram and Carmon, 1972; Moscovitch and Smith, 1979; Schieppati et al., 1984; Kaluzny et al., 1994; Fendrich et al., 2004). This high variability in the tactile CUD might reflect different type of stimulation used, or to the fact that some studies stimulate the same limb used to respond while other used different limbs.

Modulatory effects on the CUD as a function of the sensory callosal connections are provided by neuropsychological studies on patients with congenital absence of the CC or following surgical section of the CC (Volpe et al., 1982; Tassinari et al., 1994). For instance, Tassinari et al. (1994) in a simple visuomotor response task to lateralized flashes in patients with partial section of the anterior part of the CC (the splenium was intact) found that the CUD was comparable to the ones of healthy participants. As the splenium is believed to mediate transfer of visual information in both animals (Segraves and Innocenti, 1985) and humans (Saenz and Fine, 2010; Knyazeva, 2013), authors suggested that the CUD in their patients could reflect the inter-hemispheric transfer of the signal through both sensory and motor channels. Similarly, Volpe et al. (1982) studying patients that underwent partial callosotomy showed that the anterior part of the CC cannot transfer critical visual or sensorimotor information that is necessary to perform motor action (Volpe et al., 1982). The posterior CC has been identified as the pathway mediating transfer of tactile learning to the opposite hemisphere (Stamm and Sperry, 1957; Myers and Ebner, 1976). Recently, Diffusion Tensor Imaging (DTI) data in humans has shown that fibers in the posterior CC, particularly in region IV, carry somatosensory signals, whereas more anterior areas such as region III and region II are responsible for motor and premotor information transfer, respectively (Caminiti et al., 2013; Fling et al., 2013). Similarly, Fabri et al. (2001, 2005) studied patients who underwent a two stage resection of the CC, starting with the anterior CC (stage 1) followed by the posterior CC and splenium (stage 2). Their results showed that normal inter-hemispheric transfer of tactile information required the posterior CC.

Interestingly, the CUD effect was significantly greater at the most distal regions of the body (i.e., fingers and forearm) compared to a more proximal part such as the forehead (see Figure 1A). This pattern of results is compatible with the relative distribution of the callosal connections and bilateral RFs that are denser for the axial regions of the body such as the trunk, head and oral cavity and sparser for the more distal regions (Killackey et al., 1983; Conti et al., 1986; Innocenti, 1986; Iwamura, 2000). However, neurons with bilateral RFs have been found for the shoulders and arms (Taoka et al., 1998) as well as to a lesser degree for the fingers (Iwamura et al., 1994). The fact that the CUD exists on the fingers, despite the presence of some bilateral RFs may be due to the fact that these neurons respond only under particular conditions such as for instance, subsequent simultaneous or in a short delay bilateral stimulations (Tamè et al., 2015) or when hands are involved in certain actions. Indeed, these bilateral neurons may have important functional implications in tasks that required bi-manual coordination (Iwamura et al., 2001; Farnè et al., 2007). In this respect, Tamè et al. (2012, 2015), using a tactile repetition suppression (RS) paradigm, showed strong finger-specific neural responses in the primary (SI) and secondary (SII) somatosensory cortices of the hemisphere contralateral to the stimulated hand. However, the RS approach allowed these authors to unearth, though to a lesser degree than in the contralateral hemisphere, the presence of finger-specific neural activity in the ipsilateral hemisphere for the same brain areas at the early stages of the tactile information processing (Tamè et al., 2012, 2015).

A similar organization has also been found in the motor cortex. For example, experiments on split-brain monkeys have shown that each hemisphere controls the contralateral arm, hand and finger (Brinkman and Kuypers, 1973). However, the ipsilateral hemisphere mainly controls voluntary movements of more proximal part of the body such as the arm (Tazoe and Perez, 2014). Anatomical evidence has suggested that uncrossed fibers may make up 10–15% of fibers in the lateral spinal tracts in both humans (Nyberg-Hansen and Rinvik, 1963) and monkeys (Glees and Cole, 1952). However, fMRI studies in humans have shown that unilateral movement of the hand produces activation of the contralateral hemisphere about 20 times greater than corresponding regions of the ipsilateral hemisphere (Kim et al., 1993). Therefore, control of the distal movements of the foot is primarily operated by the contralateral motor cortex (Seeley et al., 1992; Ganong, 1993; Hellige, 1993).

### Interhemispheric Transfer Through Sensory, Motor or Sensorimotor Channels

Most studies in vision support a motor account for the CUD effect. For instance, it has been shown that varying physical properties of the stimulus such as luminance (Clarke and Zaidel, 1989) and retinal eccentricity (Berlucchi et al., 1971, 1977; Aglioti et al., 1991) did not modulate the CUD effect (for similar results see also Berlucchi et al., 1977, 1995; Aglioti et al., 1993; Forster and Corballis, 1998). In an intermediate position between the sensory and motor accounts, Milner and Lines (1982) measured simple RT as a function of light intensity, finding that the CUD varied with intensity when participants made vocal responses, but not when they made manual responses. Moreover, Bisiacchi et al. (1994) proposed an interhemispheric transmission process based on a horse race model in which information is transferred at both sensory and motor levels and the CUD value reflects which of the signals is fastest on a particular trial (Bisiacchi et al., 1994). In this respect, our results provide evidence suggesting the involvement of sensory channels in the interhemispheric integration. We cannot, however, rule out the possibility of the involvement of the motor channels. It is likely that in the tactile domain the sensorimotor channels jointly mediate the interhemispheric transfer of the information between the two hemispheres.

As mentioned above, our results cannot be explained by stimulus response spatial compatibility, as we controlled the spatial position of the responding foot. Moreover, Anzola et al. (1977) suggest that an anatomical account for CUD is compatible in a simple RT (as in the present work), whereas a stimulus-response spatial compatibility plays a more relevant role in a choice situation. In this respect, Mooshagian et al. (2009) have shown that in the absence of the CC and complete commissurotomy non-anatomical factors can affect CUD (Mooshagian et al., 2009). More recently, Pellicano et al. (2013) ruled out the possibility of a stimulus-response spatial compatibility effect for the CUD in both right as well as left handed individuals (Pellicano et al., 2013).

Finally, previous studies have shown that the physical distance (Shore et al., 2005) at which the tactile stimuli are presented on the two sides of the body and the relative position of the body in space (Tamè et al., 2011) can affect participant’s performance. Even though in the present work tactile stimuli on the forehead were closer (i.e., 2 cm) compared to the fingers (i.e., 20 cm) and forearms (i.e., 30 cm) the results cannot be explained by a spatial modulation effect because although the stimuli on the forearms were more distant than stimuli on the fingers we did not find a significant CUD for the forearm.

### CUD Asymmetry is not Modality and Limb Specific

In the trials in which participants responded with their left foot they were significantly faster for the uncrossed than crossed condition (i.e., positive CUD) compared to when they responded with the right foot. With right foot responses there was a trend towards a negative CUD effect, though this did not reach significance. This asymmetry, mirrors the results of Fendrich et al. (2004), who found positive tactile and visual CUDs for left-handed, but not for right-handed responses. Another study using electrical stimulation at the fingers reported a greater CUD when responses were made with the left finger than with the right finger (Kaluzny et al., 1994). Further, a meta-analysis on 16 studies conducted by Marzi et al. (1991) showed that a positive CUD is present when participant respond with the left hand, whereas a negative CUD emerged when participant respond with their right hand. Marzi and colleagues suggested that this asymmetry might reflect faster transfer of signals from the right to the left than from the left to the right hemisphere (Marzi et al., 1991; Marzi, 2010; Pellicano et al., 2013). An alternative explanation of the differences we found between the CUDs when the left or right foot was used to respond could derive, at least in part, from the fact that participants responded faster to the preferred (i.e., right hand) compared to the non-preferred (i.e., left hand) hand.

## Conclusion

The present results show a modulation of the tactile CUD as a function of the body part stimulated. A greater CUD effect was present for stimuli on the fingers and forearm compared to stimuli on the forehead, compatible with denser callosal connections between regions that represent the most proximal parts of the body relative to regions that represent the extremities. This suggests that the interhemispheric transfer of tactile stimuli is modulated by the callosal connections of the stimulated body regions. Finally, the CUD asymmetry we reported is in agreement with previous reports from similar studies and extend them by showing the presence of this effect with tactile stimuli when foots are used to respond.

## Conflict of Interest Statement

The authors declare that the research was conducted in the absence of any commercial or financial relationships that could be construed as a potential conflict of interest.
